# Hypothalamic volume loss is associated with reduced melatonin output in Parkinson's disease

**DOI:** 10.1002/mds.26592

**Published:** 2016-03-12

**Authors:** David P. Breen, Cristina Nombela, Romina Vuono, P. Simon Jones, Kate Fisher, David J. Burn, David J. Brooks, Akhilesh B. Reddy, James B. Rowe, Roger A. Barker

**Affiliations:** ^1^ John van Geest Centre for Brain RepairUniversity of CambridgeCambridgeUK; ^2^ Department of Clinical NeurosciencesUniversity of CambridgeCambridgeUK; ^3^ Institute of NeuroscienceNewcastle UniversityNewcastleUK; ^4^ Division of Neurology, Imperial CollegeLondonUK; ^5^ Institute of Clinical MedicineAarhus UniversityAarhusDenmark; ^6^ Institute of Metabolic ScienceUniversity of CambridgeCambridgeUK; ^7^ Behavioural and Clinical Neuroscience InstituteUniversity of CambridgeCambridgeUK; ^8^ Medical Research Council Cognition and Brain Sciences UnitCambridgeUK; ^9^ Present address: School of Medicine, Universidad Politécnica de CartagenaMurciaSpain; ^10^ Present address: School of Chemistry, University of EdinburghEdinburghUK

**Keywords:** melatonin, hypothalamus, suprachiasmatic nucleus, Parkinson's, circadian

## Abstract

**Background:**

Recent studies have suggested that melatonin—a hormone produced by the pineal gland under circadian control—contributes to PD‐related sleep dysfunction. We hypothesized that degenerative changes to the neural structures controlling pineal function (especially the suprachiasmatic nuclei of the anterior hypothalamus) may be responsible for reduced melatonin output in these patients. We compared hypothalamic volumes in PD patients with matched controls and determined whether volume loss correlated with reduced melatonin output in the PD group.

**Methods:**

A total of 12 PD patients and 12 matched controls underwent magnetic resonance imaging to determine hypothalamic volume. In addition, PD patients underwent 24‐hour blood sampling in a controlled environment to determine serum melatonin concentrations using enzyme‐linked immunosorbent assays.

**Results:**

PD patients had significantly reduced hypothalamic gray matter volume when compared with matched controls. Melatonin levels were significantly associated with hypothalamic gray matter volume and disease severity in PD patients.

**Conclusion:**

Melatonin levels are associated with hypothalamic gray matter volume loss and disease severity in PD patients. This provides anatomical and physiological support for an intrinsic sleep and circadian phenotype in PD. © 2016 The Authors. Movement Disorders published by Wiley Periodicals, Inc. on behalf of International Parkinson and Movement Disorder Society

Sleep disturbances are one of the most common nonmotor complaints in Parkinson's disease (PD) and have been attributed to a variety of factors. Understanding the relative contribution of each is crucial to identify the most effective treatment strategies for individual patients. Some of these relate to the clinically identified features of the disease such as motor impairment, nocturia, pain, or neuropsychiatric symptoms. Dopaminergic and other medications may also exacerbate patients' sleep problems. However, the sleep dysfunction in PD may be a result of neuronal loss in key structures and circuits involved in the regulation of the sleep‐wake cycle.

Two recent studies have reported that reduced melatonin output in PD patients is associated with altered sleep architecture, including reduced slow wave and rapid eye movement sleep^1^ and excessive daytime sleepiness.^2^ Altered melatonin patterns have also been observed in Huntington's disease^3^ and Alzheimer's disease,^4^ both of which have prominent sleep and circadian abnormalities. Because melatonin is a hormone produced by the pineal gland under circadian control, we hypothesised that degenerative changes to the neural structures controlling pineal function (especially the suprachiasmatic nuclei [SCN] of the hypothalamus) may reduce melatonin output and contribute to certain aspects of sleep dysfunction in PD.

This study compared hypothalamic volumes in PD patients with matched controls and determined whether volume loss correlated with reduced melatonin output in the PD group.

## Methods

### Patients

A total of 12 PD patients were selected from a previously studied sleep cohort.^1^ All of the patients who had also undergone magnetic resonance imaging (MRI) as part of the parallel Incidence of Cognitive Impairment in Cohorts with Longitudinal Evaluation–Parkinson's Disease (ICICLE‐PD) study were included in the analysis, in addition to 12 unrelated matched controls from the Medical Research Council Cognition and Brain Sciences Unit healthy volunteer panel. The ICICLE‐PD protocol has been published elsewhere.^5^ All participants provided written consent, the study was performed according to the Declaration of Helsinki, and the protocol was approved by the local research ethics committee.

In brief, patients underwent a battery of clinical tests including the Unified Parkinson's Disease Rating Scale (MDS‐UPDRS), Addenbrooke's Cognitive Examination (ACE‐R), and the Beck Depression Inventory (BDI). Levodopa equivalent dose (LED) was calculated using the conversion factors proposed by Tomlinson and colleagues.^6^ Matching was based on age, gender, years of education, and ACE‐R.

### Imaging Acquisition and Analysis

**Figure 1:**
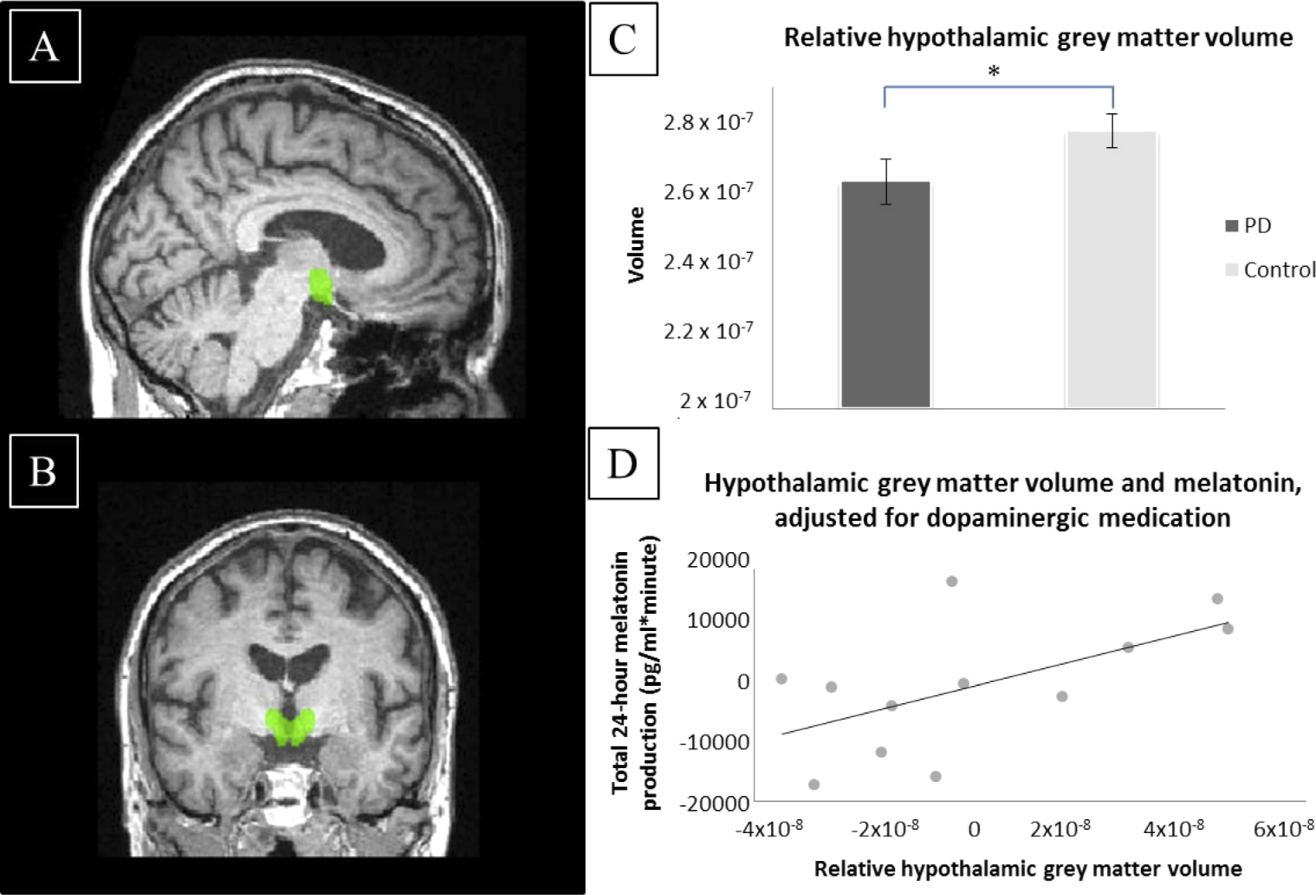
**A** and **B**: Panels show the region of interest used to calculate the hypothalamic volume for each participant. **C**: Panel is a graphical representation of the significant reduction in relative hypothalamic gray matter volume in PD patients when compared with matched controls (with standard error of the mean error bars). **D**: Panel demonstrates the significant correlation between relative hypothalamic gray matter volume and total 24‐hour melatonin output (with both axes showing partial residuals). In both graphs, relative hypothalamic gray matter volume was calculated by dividing gray matter volume by whole brain volume (both measured in voxels). [Color figure can be viewed in the online issue, which is available at wileyonlinelibrary.com.]

MRI data were acquired using a Siemens TIM Trio 3T scanner (Siemens Medical Systems, Erlangen, Germany). Patients underwent T1‐weighted magnetisation prepared rapid gradient echo scanning: pulse repetition = 2250 ms, echo time = 2.98 ms, flip angle = 9 °, time delay = 900 ms, 256 × 256 mm^2^ field of view, 192 × 1 mm slices). Images were preprocessed according to a pipeline in SPM8 (http://www.fil.ion.ucl.ac.uk/spm) run on Matlab 7 (Mathworks, Natick, Massachusetts). T1‐weighted images were segmented into gray matter and white matter tissue and registered through the Diffeomorphic Anatomical Registration Through Exponentiated Lie Algebra scheme. The resulting study‐specific template was registered to Montreal Neurological Institute space, and individual modulated images were smoothed with an 8 mm, full width at half‐maximum Gaussian kernel. A hypothalamic region of interest (dilated by 3 mm) from the WFU Pick Atlas (http://fmri.wfubmc.edu/software/pickatlas) was used to obtain an individual hypothalamic volume per participant (Fig. 1A,B). Gray matter volume in the region of interest (measured in voxels) was calculated using the FMRIB Software Library (FSL) tool “fslstats” within FSL version 4.1.7 (www.fmrib.ox.ac.uk). Thereafter, relative hypothalamic gray matter volume was calculated by dividing by whole brain volume (the sum of the gray and white matter segments).

### Serum Melatonin Measurement

PD patients were admitted to a single room at the Wellcome Trust Clinical Research Facility at Addenbrooke's Hospital in Cambridge. A peripheral venous cannula was inserted 30 minutes before the start of sampling at 1:00 pm. During the next 24 hours, participants adhered to their habitual bed times, and blood samples were collected every 90 minutes using a 3‐way valve that was attached to a 0.9% sodium chloride infusion to prevent the cannula from clotting. Sampling was performed through a long line to prevent disruption to the patient's sleep. Participants remained sedentary apart from bathroom visits. Meal times were consistent between participants, and no daytime naps were allowed. Temperature was constant at 21 °C. Patients were not strictly shielded from external light, but lighting levels were less than 5 lux following lights off. Serum melatonin concentrations were measured using enzyme‐linked immunosorbent assays as previously described.^1^ Based on hormone concentrations at each 90‐minute time point, total 24‐hour melatonin output was defined as the area under the curve (calculated using the trapezoid rule).

### Statistical Analysis

All data were approximately normally distributed based on Shapiro‐Wilk testing; therefore, unpaired *t* tests were used to compare clinical parameters and volumetric values between patients and controls. Pearson rank correlation testing was used to study the relationship between melatonin output and relative hypothalamic gray matter volume, as well as the relationship between melatonin output and disease severity (adjusted for LED).

## Results

Age, gender, duration of education, and ACE‐R were not significantly different between PD patients and controls (Table 1). PD patients had a mean disease duration of 3.3 years, mean LED of 366 mg, and mean UPDRS part III score of 23.9. None of the participants were taking hypnotics. The mean duration between melatonin testing and MRI in the PD group was 1.9 months (SD 3.4).

**Table 1:** Clinical characteristics of PD patients and controls

Variable	PD	Controls	*P* value^d^
Number of participants	12	12	na
Gender ratio (male:female)	6:6	6:6	1.0
Age (years)	66.7 (5.5)	66.3 (5.2)	0.92
Duration of education (years)	18.3 (2.9)	17.2 (2.8)	0.33
ACE‐R	95.2 (3.1)	96.1 (3.0)	0.48
Disease duration (years)^a^	3.3 (1.1)	na	na
LEDD (mg)^b^	366 (161)	na	na
MDS‐UPDRS part III^c^	23.9 (9.0)	na	na
BDI	7.3 (17.8)	3.3 (3.6)	0.011*

*Significant difference at .05 level.

LEDD, levodopa equivalent daily dose; ACE‐R, Addenbrooke's Cognitive Examination‐Revised; MDS‐UPDRS, Unified Parkinson's Disease Rating Scale; BDI, Beck Depression Inventory; na, not applicable.

Results expressed as mean (SD) unless stated otherwise

Significant difference at .05 level.

a Disease duration from date of diagnosis.

b All but two PD patients were taking dopaminergic medication.

c Based on MDS‐UPDRS assessments performed within the last 6 months.

d Unpaired *t* tests performed.

When compared with controls, PD patients had significantly reduced relative hypothalamic gray matter volume (2.56 × 10^−7^ [SD 2.78 × 10^−7^] vs 2.69 × 10^−7^ [SD 2.07 × 10^−7^]; *P* = .005) (Fig. 1C).

Having verified that there were significant differences between patients and controls in terms of hypothalamic volume, we found that melatonin levels were significantly associated with relative hypothalamic gray matter volume in the PD group (*r* = .591, *P* = .028) (Fig. 1D).

Partial correlation between melatonin levels and disease severity, correcting for LED, showed a significant inverse relationship (*r* = −.681, *P* = .021). There was no significant relationship between melatonin output and LED (*r* = .180, *P* = .76).

## Discussion

There is increasing evidence from clinical and animal studies that there is circadian dysregulation in a variety of neurodegenerative diseases.^7^ We previously reported significant reductions in melatonin concentration in 30 early‐stage PD patients.^1^ Videnovic and colleagues also found a significantly diminished amplitude of melatonin secretion in serum samples of 20 PD patients on dopaminergic therapy under modified constant routine conditions.^2^

There is evidence from neuropathological^8^ and imaging^9^ studies that the hypothalamus is directly affected by PD. The central clock within the hypothalamus, the SCN, is likely to contribute to this volume loss because it has been shown that mice overexpressing alpha‐synuclein exhibit a reduced SCN firing rate.^10^ This could weaken their ability to communicate neural and hormonal signals from the central clock to the pineal gland, which secretes melatonin into the blood.

This study thus adds to the existing literature by suggesting that hypothalamic volume loss—which we have now shown in this new PD cohort—may be responsible for reduced melatonin output, which has been linked to sleep disturbances in PD.

The major limitation of this study is the relatively small number of patients, which precluded the use of linear regression and adjustment of confounders. Furthermore, patients were not strictly shielded from external light during the melatonin sampling period, which may have influenced the results. Although we lacked serum melatonin measurements in the control group, the critical test for our hypothesis was the correlation between hypothalamic volume and melatonin levels in PD patients. It is not yet possible to perform dedicated imaging of the SCN within the hypothalamus using 3Tesla MRI; therefore, ultra‐high field imaging or clinico‐pathological studies will be required to allow more thorough dissection of the relative role of the different hypothalamic nuclei to this deficit.

In summary, we have shown that melatonin levels are associated with hypothalamic gray matter volume loss and disease severity in PD patients. This provides anatomical and physiological support for an intrinsic sleep and circadian phenotype in PD and the fact that this is related to the disease itself rather than being an indirect consequence of other symptoms or treatments.

## Author Roles

(1) Research Project: A. Conception, B. Organization, C. Execution; (2) Statistical Analysis: A. Design, B. Execution, C. Review and Critique; (3) Manuscript: A. Writing of the First Draft, B. Review and Critique.

D.P.B.: 1A, 1B, 1C, 2C, 3A

C.N.: 2B, 2C, 3A

R.V.: 2B, 3B

P.S.J.: 2A, 2C, 3B

K.F.: 2B, 3B

D.J. Burn: 3B

D.J. Brooks: 3B

A.B.R.: 2C, 3B

J.B.R.: 2C, 3B

R.A.B.: 3B

## Full financial disclosures of all authors for the past 12 months

D.P.B. has received speaker fees from UCB Pharma. C.N. has received institutional support from the University of Cambridge for the Incidence of Cognitive Impairment in Cohorts with Longitudinal Evaluation–Parkinson's Disease project (funded by Parkinson´s UK) and from the Technical University of Cartagena (Murcia, Spain) for the EXO‐LEGS project (funded by the Active Assistance Living European programme). D.J. Burn has received institutional research funding support from the National Institute of Health Research, Wellcome Trust, Parkinson's UK, and Michael J Fox Foundation; funding support from the Newcastle National Institute of Health Research Biomedical Research Unit; and speaker fees from Acadia Pharmaceuticals. D.J. Brooks is a consultant for GE Healthcare and has been paid honoraria by them for lecturing, he has served on advisory boards for Astex and GenePod, and he has been paid honoraria by Brittania, Zambon, and Astra Zeneca. J.B.R. has received an honorarium from Lilly for teaching and research grant support from AZ‐Medimmune. R.A.B. has received royalty payments from Springer for editorial work, royalty payments from Wiley for medical textbooks, and consultancy payments from Living Cell Technologies over their cell therapy program for Parkinson's disease. R.V., K.F., S.J., and A.B.R. report no disclosures.
